# Clinical study of interstitial brachytherapy for 72 cases of recurrent cervical cancer

**DOI:** 10.12669/pjms.39.3.6868

**Published:** 2023

**Authors:** Yunfeng Guo, Ge Jin, Yang-yang Gao, Kuixiu Li

**Affiliations:** 1 Yunfeng Guo, Department of Gynecology and Oncology, The Fourth Hospital of Hebei Medical University, Shijiazhuang 050000, Hebei, P.R. China; 2 Ge Jin, Department of Gynecology and Oncology, The Fourth Hospital of Hebei Medical University, Shijiazhuang 050000, Hebei, P.R. China; 3 Yang-yang Gao, Department of Oncology, Liaocheng Tumor Hospital, Liaocheng 252002, Shandong, P.R. China; 4 Kuixiu Li, Department of Gynecology and Oncology, The Fourth Hospital of Hebei Medical University, Shijiazhuang 050000, Hebei, P.R. China

**Keywords:** Recurrence, Cervical cancer, Interstitial brachytherapy, Toxicity and side effects, Prognosis

## Abstract

**Objective:**

To determine the application value of interstitial brachytherapy in the treatment of recurrent cervical cancer.

**Methods:**

A retrospective analysis was conducted on the clinical data of 72 patients with recurrent cervical cancer admitted to The Fourth Hospital of Hebei Medical University from September 2017 to April 2022. They were divided into two groups according to different brachytherapy methods: conventional after-load radiotherapy group and interstitial brachytherapy group. After treatment, regular outpatient reviews or telephone follow-ups were conducted to evaluate the efficacy, related toxic and side effects and prognostic factors.

**Results:**

The short-term efficacy of the interstitial brachytherapy group was significantly higher than that of the interstitial brachytherapy group (p<0.05). The one-year LC and two-year LC of the interstitial brachytherapy group were 94% and 90.6%, respectively, while those of the conventional after-load group were 74.5% and 67.8%, respectively, with a statistically significant difference (p<0.05). The clinical efficacy of peripheral recurrence was 13.9% in the interstitial brachytherapy group, and that in the conventional after-load group was 2.7%, with a statistically significant difference (p<0.05). There was a statistically significant difference in late toxic and side effects between the two groups (p<0.05). Prognostic factors: Multivariate analysis of the COX regression model showed that only the maximum tumor diameter was an independent prognostic factor for OS and PFS, while the recurrence site and brachytherapy method were the independent prognostic factors for LC.

**Conclusion:**

Interstitial brachytherapy radiotherapy touts various benefits in the treatment of patients with recurrent cervical cancer, such as good short-term efficacy, high local control rate, reduced incidence of advanced bladder and rectal toxicity, and improved quality of life.

## INTRODUCTION

According to the International Federation of Gynecology and Obstetrics (FIGO), the recurrence rate of patients with stage IB, IIA, IIB, III and IVA cervical cancer who received radiotherapy alone was 10%, 17%, 23%, 42% and 74%, respectively.^1^,^2^ Cervical cancer is less likely to be treated again if it recurs after treatment. It has been reported that patients with recurrent cervical cancer have a poor prognosis, with a one year survival rate of 15%-20% and five-year survival rate of 3.2%-16.5%.^3^,^4^ Local recurrence is the most common cause of death in cervical cancer, and for recurrent cervical cancer, treatment options such as surgery, radiotherapy, and chemotherapy are preferred. However, pelvic debridement has low resection rate, high incidence of postoperative complications and poor postoperative quality of life. Palliative chemotherapy has a limited effect on prolonging the median survival of recurrent cervical cancer. In patients with recurrent, persistent and metastatic cervical cancer, chemotherapy combined with bevacizumab can only prolong overall survival by 3.7 months.^5^,^6^ Recurrent cervical cancer is a very difficult problem in clinical practice. Currently, three-dimensional interstitial brachytherapy alone or combined with external-beam imRT has been gradually applied in the treatment of recurrent cervical cancer at home and abroad, but its efficacy and safety are still under further exploration. In order to investigate the application value of interstitial brachytherapy in the treatment of recurrent cervical cancer, in this paper, the efficacy, toxic and side effects and prognostic factors of interstitial brachytherapy and conventional after-load radiotherapy were compared and analyzed in the treatment of recurrent cervical cancer, so as to provide a basis for the formulation of the treatment regimen for recurrent cervical cancer.

## METHODS

A retrospective analysis was used in this study. A total of 72 patients with recurrent cervical cancer admitted to the Fourth Hospital of Hebei Medical University from September 2017 to April 2022 were included and divided into two groups according to different brachytherapy methods: conventional after-load radiotherapy group and interstitial brachytherapy group, with 36 cases in each group. The sample size required for each group was calculated by the formula.


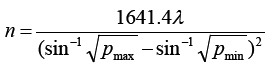


The study was approved by the Institutional Ethics Committee of The Fourth Hospital of Hebei Medical University (No.:2018MZC147; date: November 28, 2018), and written informed consent was obtained from all participants.

### Inclusion criteria:

- Patients whose treatment includes brachytherapy after recurrence;
- Patients with pathologic diagnosis of recurrence or recurrence indicated by medical history, the continuous elevation of tumor marker squamous cell carcinoma antigen (SCCA) combined with MAGNETIC resonance imaging (MRI), computed tomography (CT) and positron emission computed tomography (PET/CT);
- Patients with stage according to FIGO stage 2018; 4) Patients with KPS≥70 points.

### Exclusion criteria:

- Patients with contraindications to radiotherapy;
- Patients with serious medical diseases;
- Patients with distant metastasis.
- The general data of the two groups were comparable (see Table-I).

**Table-I:** General clinical data of 72 patients with recurrent cervical cancer.

Data of cases	Interstitial brachytherapy (n=36)	Conventional after-load radiotherapy (n=36)	p
Median age of recurrence (years old)	51 (28-75)	54 (40-69)	0.809
Hemoglobin1 (g/L)	120.72±24.05	126.43±11.91	0.206
***Recurrence interval (months)***			0.437
≤24	27	24	
>24	9	12	
***FIGO staging***			0.077
IB-IIA	21	28	
IIB-III	15	8	
SCCA (ng/ml)	6.95±9.57	7.63±14.85	0.817
***Histological type***			0.743
Squamous cell carcinoma	33	35	
Adenocarcinoma	1	1	
Adenosquamous carcinoma	2	0	
***Maximum tumor diameter (cm)***			0.157
<4	14	20	
≥4	22	16	
***Previous radical surgery***			0.074
Yes	26	32	
No	10	4	
***Recurrence site***			0.551
Central type	30	28	
Peripheral type	6	8	
***Concurrent chemoradiotherapy***			0.496
Yes	30	32	
No	6	4	
***Previous radiotherapy history***			0.169
Radical	10	4	
Auxiliary	2	5	
No	24	27	

Intravenous contrast media was administered to all patients for enhanced scanning and three-dimensional reconstruction. Target areas were delineated by the clinician. Based on localized CT combined with clinical physical examination, MRI or PET/CT after recurrence, first, gross tumor volume (GTV) and clinical target volume (CTV) were delineated, including the recurrence lesion, vagina, pelvic cavity and some para-aortic lymph node drainage areas. In part of the para-aortic lymph node drainage area, the CTV was expanded by 0.6 cm in the anterior-posterior, left-right, and lateral directions, and evenly expanded by one cm in the upper and lower directions to obtain the planning target volume (PTV). After that, a radiation therapy plan was designed by a physicist, which requires 95% of the volume of PTV to meet the prescribed dose and the limit for OAR. External-beam radiation was initiated after the completion of the radiotherapy plan.

According to the gynecological physical examination, the recurrence site, and the regression of the recurrent tumor detected by CT/MRI after three-four weeks of external-beam irradiation (radiotherapy dose of 27-36 Gy), the path, depth and number of needles for needle insertion are preliminarily determined. Under the guidance of CT, the insertion depth was adjusted to determine whether additional insertion needles were needed. After the operation, the high-risk clinical target volume (HR-CTV) and OARs were delineated. A physicist was assigned to perform the registration, fusion and reconstruction of the applicator, set a dose of D90 6-7 Gy for each prescription, and design a treatment plan for treatment according to the above requirements.

### Conventional after-load radiotherapy:

A gynecological examination was performed before the applicator was placed, and the appropriate applicator was selected based on the patient’s recurrence site and CT and MRI. In each case, five mm of 6-7 Gy was administered at point A or subvaginal mucosa.

The curative effect was evaluated according to the MRI or CT examination combined with gynecological examination three months after the end of recurrence treatment, and the evaluation standard was based on RECIST1.1 (Response Evaluation Criteria in Solid Tumors). Complete response (CR): all target lesions disappeared for at least 4 weeks; Partial response (PR): lesions shrink by at least 30% for at least four weeks; Stable disease (SD): between PR and PD; PD: lesions increase by more than 20%, or new lesions appear. The toxicity assessment was based on the Radiological injury classification of the Radiation Therapy Oncology Group (RTOG).These patients were followed up for 6 months postoperatively.

All data in this study were analyzed using SPSS 21.0. T test was used for measurement data, the Chi-square test was employed for counting data, and the Kaplan-Meier method was utilized to calculate OS, PFS and LC and draw survival curve. Univariate prognostic analysis was performed by log-rank and multivariate analysis was performed by COX proportional risk regression model. P<0.05 indicates a statistically significant difference.

## RESULTS

The clinical effective rate of the interstitial brachytherapy group was 91.6%, which was significantly higher than the conventional after-load radiotherapy group (p<0.05). Table-II.

**Table-II:** Comparison of short-term efficacy.

Efficacy	Interstitial brachytherapy (n=36)	Conventional after-load radiotherapy (n=36)	c^2^	p
CR+PR	33	26	4.6	0.032
CR	27	23		
PR	6	3		
SD	1	4		
PD	2	6		

**Fig.1:**
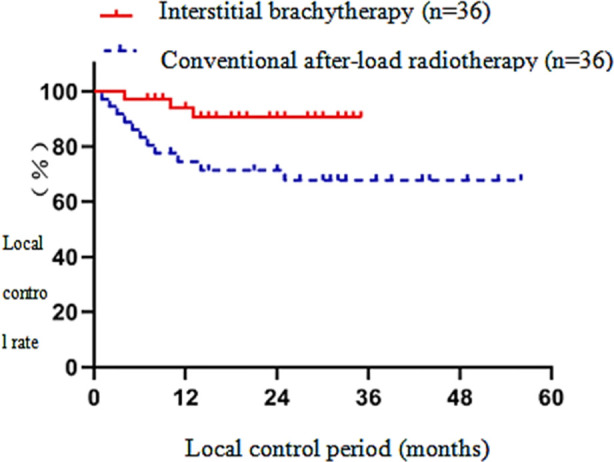
Local control survival curve of interstitial brachytherapy and conventional after-load radiotherapy.

**Fig.2:**
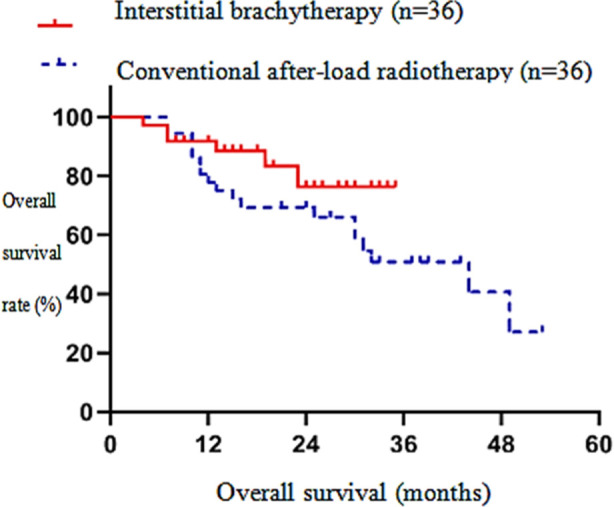
Overall survival curve of interstitial brachytherapy and conventional after-load radiotherapy.

**Fig.3:**
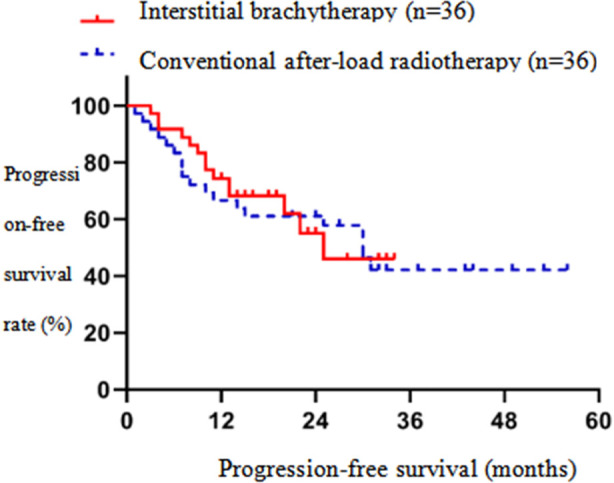
Progression-free survival curve of interstitial brachytherapy and conventional after-load radiotherapy.

The one-year LC and two-year LC of the interstitial brachytherapy group were significantly higher than those of the conventional after-load radiotherapy group (p<0.05). Fig.1 In the interstitial brachytherapy group, the one-year OS and two-year OS were significantly higher than those of the conventional after-load radiotherapy group (p<0.05).Fig.2 In the interstitial brachytherapy group, the one-year PFS and two-year PFS were 74.4% and 55.1%, respectively, while in the conventional after-load radiotherapy group, the one-year PFS and two-year PFS were 66.7 % and 57.9%, respectively, with a statistically significant difference (p<0.05).Fig.3 The clinical effective rate of peripheral type recurrence in the interstitial brachytherapy group was significantly higher than that of the conventional after-load radiotherapy group (p<0.05).Table-III

**Table-III:** Comparison of short-term efficacy at recurrence sites.

Recurrence sites	Efficacy	Interstitial brachytherapy	Conventional after-load radiotherapy	p
Central type	CR+PR	28	25	0.665
	SD+PD	2	3	
Peripheral type	CR+PR	5	1	0.026
	SD+PD	1	7	

The early toxic and side effects of bladder, rectum and bone marrow were compared between the two groups, with no statistically significant differences (p>0.05).Table-IV The incidence of late toxicities in the interstitial brachytherapy group was significantly lower than that in the conventional after-load radiotherapy group (p<0.05). Table-V

**Table-IV:** Comparison of early toxic and side effects.

Organ	Grading	Interstitial brachytherapy (n=36)	Conventional after-load radiotherapy (n=36)	c^2^	p
Badder	≥ grade 2	5	8	0.845	0.358
	Grade 0-1	31	28		
Rectum	≥ grade 2	9	11	0.277	0.599
	Grade 0-1	27	25		
Bone marrow	≥ grade 2	23	27	1.047	0.306
	Grade 0-1	13	9		

**Table-V:** Comparison of late toxic and side effects.

Organ	Grading	Interstitial brachytherapy (n=36)	Conventional after-load radiotherapy (n=36)	c^2^	p
Badder	≥ Grade 2	6	14	4.413	0.035
	Grade 0-1	30	22		
Rectum	≥ Grade 2	4	12	5.143	0.023
	Grade 0-1	32	24		

The results of Log-rank univariate analysis showed that the recurrence site (p=0.007), the maximum tumor diameter (p=0.003) and hemoglobin value (p=0.016) were statistically significant, which were related to OS. There were significant differences in recurrence site (p=0.001) and maximum tumor diameter (p=0.001), which were correlated with PFS. The brachytherapy method (p=0.028), recurrence site (p< 0.001), and maximum tumor diameter (p=0.001) were statistically different, which were correlated with LC. Table-VI

**Table-VI:** Univariate analysis of prognosis of 72 cases of recurrent cervical cancer.

	OS			PFS			LC		

Factors	One year (%)	Two years (%)	p	One year (%)	Two years (%)	p	One year (%)	Two years (%)	p
Brachytherapy method			0.15			0.821			0.028
Interstitial brachytherapy	88.5	76.4		74.4	55.1		94	90.6	
Conventional after-load radiotherapy	77.8	66		66.7	57.9		74.5	67.8	
Recurrence site			0.007			0.001			<0.001
Central type	87.7	77.8		75.7	64.2		89.1	0	
Pelvic wall type	70.1	45.5		40.8	0		55.1	0	
Maximum tumor diameter (cm)			0.003			0.001			0.015
<4	94.1	85.4		85.3	77.9		91.1	0	
≥4	75.1	58.4		57	31.6		74.3	62.1	
Recurrence interval (months)			0.867			0.45			0.894
≤24	81.8	73.3		68.5	54.9		84.1	77.2	
>24	80.2	62.1		70.6	56.6		84.7	78.9	
Recurrent SCCA (ng/ml)			0.998			0.102			0.534
≤2.45	85.8	75.9		74.6	53.5		85.9	82.5	
>2.45	83	71.3		66.7	39.6		82.6	72.8	
Hemoglobin (g/L)			0.016			0.061			0.809
≤126	75	57.2		59.1	46.5		80.7	0	
>126	94.1	85.9		82.7	64.3		88.4	77.4	
FIGO staging			0.094			0.175			0.288
IB-IIA	90.2	76.2		76.5	61.7		82.3	74.6	
IIB-III	69.4	57.8		56.3	37.5		89.6		
Concurrent chemotherapy			0.339			0.442			0.48
Yes	85.3	75.1		72.8	57.9		83.7	76.3	
No	77.8	53.3		55.6	41.7		87.5	0	

COX multivariate analysis showed that maximum tumor diameter was an independent prognostic factor for OS, maximum tumor diameter was an independent prognostic factor for PFS, and recurrence site and brachytherapy method were independent prognostic factors for LC. Table-VII

**Table-VII:** Multivariate analysis of prognosis of 72 cases of recurrent cervical cancer.

Factors	OS		PFS		LC	

	p	HR (95%CI)	p	HR (95%CI)	p	HR (95%CI)
Maximum tumor diameter	0.046	2.877 (1.021-8.106)	0.018	2.859 (1.194-6.842)	0.300	2.363 (0.465-12.004)
Recurrence site	0.312	1.691 (0.611-4.675)	0.082	2.115 (0.910-4.916)	0.012	5.622 (1.465-21.571)
Brachytherapy method	0.046	2.726 (1.019-7.291)	0.202	1.639 (0.767-3.504)	0.020	4.927 (1.288-18.841)
Hemoglobin	0.029	0.368 (0.150-0.903)	0.157	0.587 (0.281-1.228)	0.707	0.804 (0.258-2.504)

## DISCUSSION

Recurrent cervical cancer is a challenging problem in clinical practice, with great difficulty in treatment and poor prognosis. Radiotherapy plays a pivotal role in the treatment of recurrent cervical cancer.^7^ In this study, 72 patients with recurrent cervical cancer were retrospectively analyzed, and it was found that interstitial brachytherapy for recurrent cervical cancer patients had the advantages of good short-term efficacy, high local control rate, reduced incidence of late bladder and rectal toxicity, and improved quality of life. Da Silva et al.^8^ treated 45 patients with recurrent cervical cancer with interstitial brachytherapy, and a good curative effect was achieved. Ye Weijun et al.^9^ reported that the efficacy of interstitial brachytherapy for recurrent cervical cancer was better than that of conventional after-load radiotherapy. Umezawa R et al.^10^ treated 18 patients with recurrent cervical cancer with interstitial brachytherapy, of which 12 cases achieved CR, with a CR rate of 67%, and the two-year LC, PFS and OS of 51.3%, 20%, 60.8%, respectively, showing good efficacy. In our study, in the interstitial brachytherapy group, the short-term efficacy was better than that of conventional radiotherapy (91.6% vs.72.2%), and the local control rate was also better than that of conventional after-load radiotherapy, which was consistent with the above research findings. In view of this, interstitial brachytherapy is preferred for the treatment of recurrent cervical cancer, boasting good short-term efficacy and local control.

Pelvic recurrence of cervical cancer can be divided into central type and peripheral type, with the former being more common.^11^ Patients with central type recurrence can be treated with pelvic exenteration or radiotherapy, but its postoperative mortality rate is as high as 13%-64%.^12^ In regard to the treatment of peripheral-type recurrent cervical cancer, a combination of radiotherapy and chemotherapy is required. Gangopadhyay A et al.^13^ investigated the causes of recurrence after radiotherapy for cervical cancer and their findings showed that the recurrence of pelvic lateral wall in patients with locally advanced cervical cancer after radiotherapy was related to the prescribed dose to the pelvic wall. Besides, increasing the parametrial dose by interstitial implantation during initial radiotherapy may prolong the median disease-free survival time. Peripheral-type recurrence is biologically similar to central-type recurrence, but its prognosis tends to be worse.^14^ The site of recurrence is an important factor affecting the prognosis of recurrent cervical cancer.^15^ And the reason why the recent efficacy of patients with peripheral-type in this study was significantly higher than that of conventional after-load radiotherapy using interstitial implantation radiotherapy (13.9% vs. 2.7%) is that interstitial implantation radiotherapy utilizes radiophysical characteristics to achieve tumor elimination by direct insertion of the applicator into the tumor area to increase the biologically effective dose to the tumor, which is not possible to be achieved with conventional after-load radiotherapy. Hence, interstitial implantation radiotherapy provides an effective treatment for patients with peripheral-type recurrence.

Interstitial brachytherapy determines the dose of organs at risk by irradiating the organ at risk with the lowest dose of 5cc, 2cc, 1cc, and 0.1cc, and the most commonly used is D2cc.^16^ Mahantshetty U et al.^17^ showed that in 30 patients with recurrent cervical cancer treated with interstitial brachytherapy, the incidence of late toxic side effects of grade two or above was 20%. Three-dimensional brachytherapy boasted improved local control rates and half of the toxicities compared with 2D brachytherapy.^18^,^19^ By delineating the organs at risk, interstitial brachytherapy can strictly limit the organs at risk to obtain a more accurate dose of the organs at risk, so as to properly evaluate the toxic and side effects of radiotherapy. In this study, the incidence of grade two or above toxic reactions of the main organs at risk in the interstitial brachytherapy group was significantly lower than that in the conventional after-load radiotherapy group, which was consistent with the above literature reports.

In terms of prognostic factors for recurrent cervical cancer, it includes tumor size, recurrence site, recurrence interval, radiotherapy interval, and clinical stage. It has been reported in the literature that the recurrence site in the vaginal wall or tumor diameter < three cm can achieve favorable local control after radiotherapy, and for the recurrence interval >three years, better OS and PFS can be obtained.^20^ In this study, the overall survival of recurrent tumors < four cm is higher than that of recurrent tumors≥4 cm (p=0.003). Tumor size is an independent prognostic factor affecting OS and PFS, and is also a related prognostic factor for LC. Therefore, in the treatment of recurrent cervical cancer, special attention must be paid to the tumor volume, and comprehensive treatment should be given to those with larger tumors in order to improve the curative effect. The conclusions of this study enrich the clinical data for the treatment of cervical cancer recurrence.

### Limitations of this study:

However, compared with conventional after-load radiotherapy, the follow-up time of the interstitial brachytherapy group in this study was short and the overall sample size was relatively small. In response to this, more cases and longer follow-up time are still needed to provide sufficient clinical evidence to further guide clinical individualized treatment.

## CONCLUSION

A comprehensive examination should be carried out before treatment of recurrent cervical cancer, and comprehensive treatment methods should be adopted. Interstitial radiotherapy boasts good short-term efficacy, local control rate and low late toxicity in the treatment of recurrent cervical cancer, while conventional after-load radiotherapy cannot cope with the variable tumor volume and shape.

### Authors’ Contributions:

**YG and GJ** designed this study, prepared this manuscript, and are responsible and accountable for the accuracy and integrity of the work.

**YG** collected and analyzed clinical data.

**KL** Data analysis**,** significantly revised this manuscript.
